# A Rare Pseudoaneurysm of the Popliteal Artery Induced by Acute‐Phase Rehabilitation Following Total Knee Replacement: A Case Report and Literature Review

**DOI:** 10.1111/os.70376

**Published:** 2026-08-11

**Authors:** Xiang Zhao, Haobo Wu, Jiajiang Jiang, Shigui Yan, Sihao Li

**Affiliations:** ^1^ Department of Orthopedic Surgery, 2nd Affiliated Hospital, School of Medicine Zhejiang University Zhejiang China

**Keywords:** complications, early postoperative exercise, popliteal artery, pseudoaneurysm, total knee replacement

## Abstract

**Background:**

Early rehabilitation programs are often considered essential for optimizing patient recovery after total knee replacement. Popliteal artery pseudoaneurysm, an infrequent but recognized risk during total knee replacement associated with early postoperative rehabilitation, is relatively rare.

**Case Presentation:**

We report a case of severe knee inversion deformity in a 56‐year‐old male patient with traumatic arthritis of the right knee. The man complained that the pain has gradually worsened over the past year. The patient underwent right knee replacement surgery at our hospital, using the Legacy Constrained Condylar Knee (LCCK) prosthesis. On the fourth day after the operation, the patient suddenly experienced tearing pain during the flexion movement of the knee joint. Immediate pain relief and other symptomatic treatments were administered, and vascular CT angiography (CTA) revealed a popliteal artery pseudoaneurysm. Subsequently, vascular surgery was performed to explore and surgically remove the pseudoaneurysm. The patient's postoperative symptoms had improved, and after 1 month of follow‐up, joint activity was good.

**Conclusions:**

This case emphasizes the importance of cautious postoperative rehabilitation methods for patients with severe knee joint deformities undergoing total knee replacement surgery, in order to balance the benefits of early activity and potential complications, ultimately improving patient safety and outcomes during rehabilitation.

## Graphical abstract

Early functional exercise after total knee arthroplasty (TKA) may cause popliteal artery pseudoaneurysm.

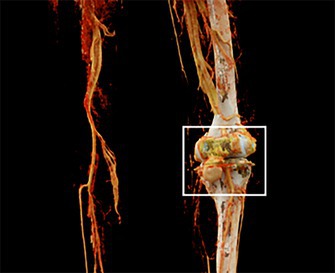

## 1 Introduction

In recent years, the prevalence of total knee replacement (TKR) procedures has surged, driven by an aging population and a growing focus on managing chronic knee pain [1]. This surgical intervention improves mobility and quality of life for many individuals; however, it is not without its complications [2]. Among the rare yet significant adverse outcomes is the development of a pseudoaneurysm in the popliteal artery [3].

Vascular injury can be catastrophic and necessitates urgent reconstruction, irrespective of whether a fasciotomy is performed. Delays in diagnosis can lead to amputation [4]. Popliteal artery injury is generally caused by two possible reasons: one is thrombosis leading to arterial occlusion, and the second is acute injury of the popliteal artery during bone cutting [5]. However, we encountered a special situation that could also be caused by early rehabilitation after surgery.

Postoperative rehabilitation protocols are critical for optimizing outcomes, focusing on restoring range of motion, improving strength, and enhancing functional abilities [6]. A multifaceted approach that includes early exercise, as highlighted in recent studies, can significantly improve recovery trajectories for patients [7, 8]. Early mobilization, paired with functional exercises, is essential for preventing complications such as stiffness and loss of functional capacity [9].

In a systematic review, researchers reported that acute‐phase rehabilitation interventions, initiated in‐hospital from immediately post‐operation to 2 weeks after surgery, showed no evidence of risk of harm, although all findings were of low strength of evidence, following total knee arthroplasty [10].

However, it is also paramount to recognize the risks associated with vigorous early rehabilitation, including potential vascular complications like pseudoaneurysms of the popliteal artery. Therefore, developing a balanced rehabilitation program that promotes recovery while minimizing risks remains a challenge that requires ongoing clinical assessment and individualized patient care. Understanding the mechanisms that contribute to this condition is crucial for clinicians engaged in post‐operative care, as it directly informs the protocols established for early rehabilitation. Consequently, the need to balance the benefits of functional exercise with the potential risks associated with premature activity becomes paramount.

In the following section, we report a case of popliteal artery pseudoaneurysm caused by early rehabilitation exercise after a TKR. The severe pain after replacement surgery made the diagnosis of pseudoaneurysm challenging, but with timely intervention, the treatment was ultimately successful.

## 2 Case Report

### 2.1 History

**FIGURE 1:**
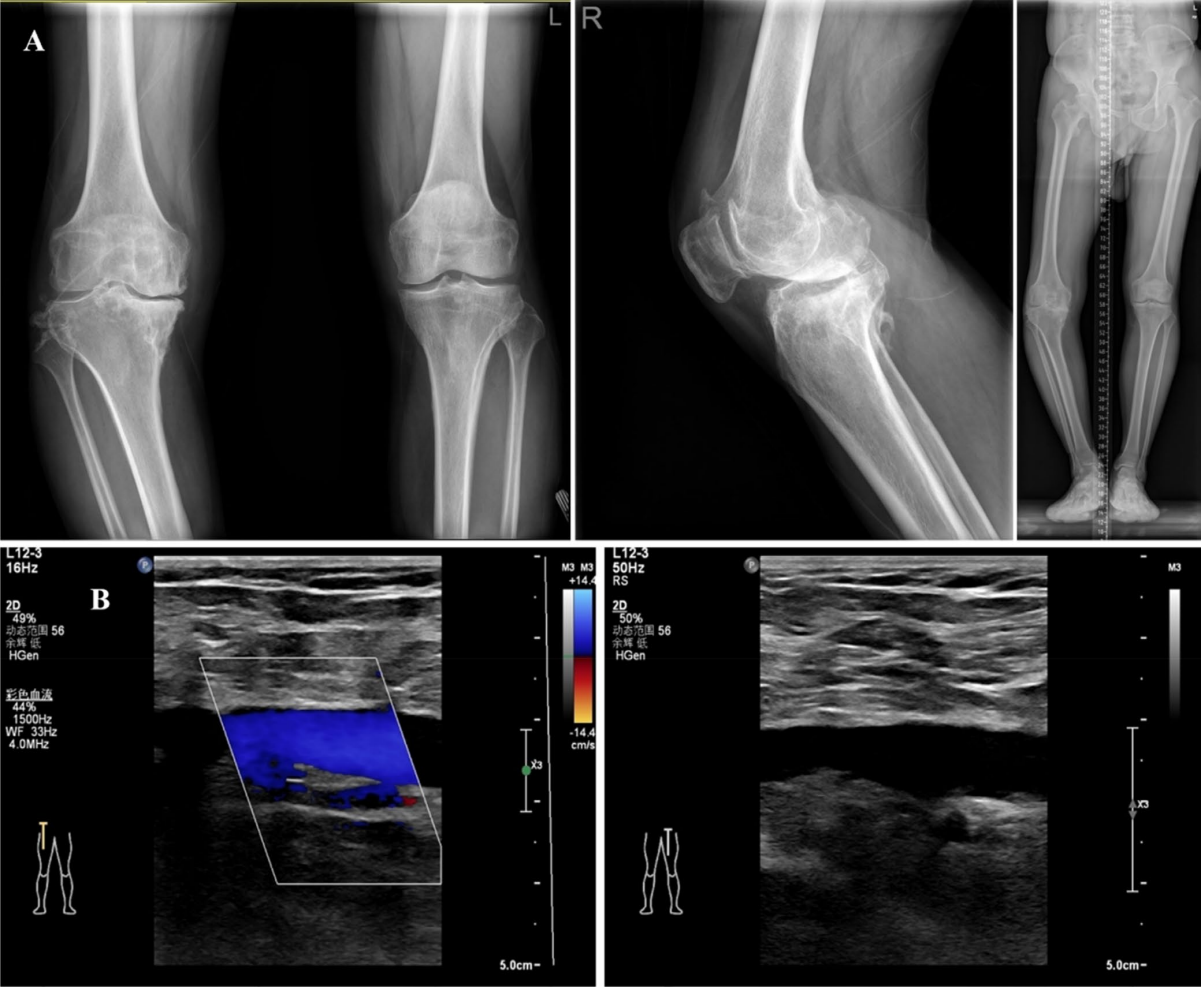
(A) Pre‐operative X‐rays of the right knee. X‐rays revealed right tri‐compartmental advanced osteoarthritic changes in the right knee, including medial joint space narrowing, subchondral sclerosis, and osteophyte formation. (B) Pre‐operative double lower limb deep vein ultrasound. The inner diameter of the deep veins in both lower limbs was normal, the endothelium was smooth, and there were no obvious abnormal echoes in the venous lumen.

The patient is a 56‐year‐old Asian male. He underwent internal fixation surgery for the tibial plateau due to a car accident 20 years ago. He has had a right knee varus deformity for over 10 years, accompanied by pain for 1 year. The patient gradually developed a right knee varus deformity 10 years ago, and 1 year ago, without any apparent cause, the patient began to experience pain, which worsened after activity and improved after rest. Upon visiting our hospital, he was diagnosed with a severe right knee varus deformity, limited mobility, and positive local tenderness. The knee joint could not be extended, with a flexion contracture of approximately 30°, and the knee joint flexion was approximately 85°, making it impossible to squat. An X‐ray of the right knee joint in the frontal and lateral positions indicated arthritis of the right knee and degenerative changes in the left knee joint (Figure 1A). Preoperative deep vein ultrasound of both lower limbs showed no significant vascular abnormalities (Figure 1B). Thus surgical treatment was recommended.

### 2.2 Surgical Technique (Right TKR)

The patient underwent a right knee replacement surgery in our hospital, using the Legacy Constrained Condylar Knee (LCCK) prosthesis. A trial model was installed, the knee joint was mobilized, and soft tissue balancing was meticulously performed. Finally, a total knee prosthesis was selected, which included a femoral size F prosthesis, a tibial Size 5 prosthesis, an 11 mm polyethylene spacer, and a patellar Size 32 prosthesis. No significant vascular damage was observed during the operation, and the intraoperative blood loss was approximately 100 mL. After achieving hemostasis, the drainage tube was inserted.

### 2.3 Postoperative Hospital Course

**FIGURE 2:**
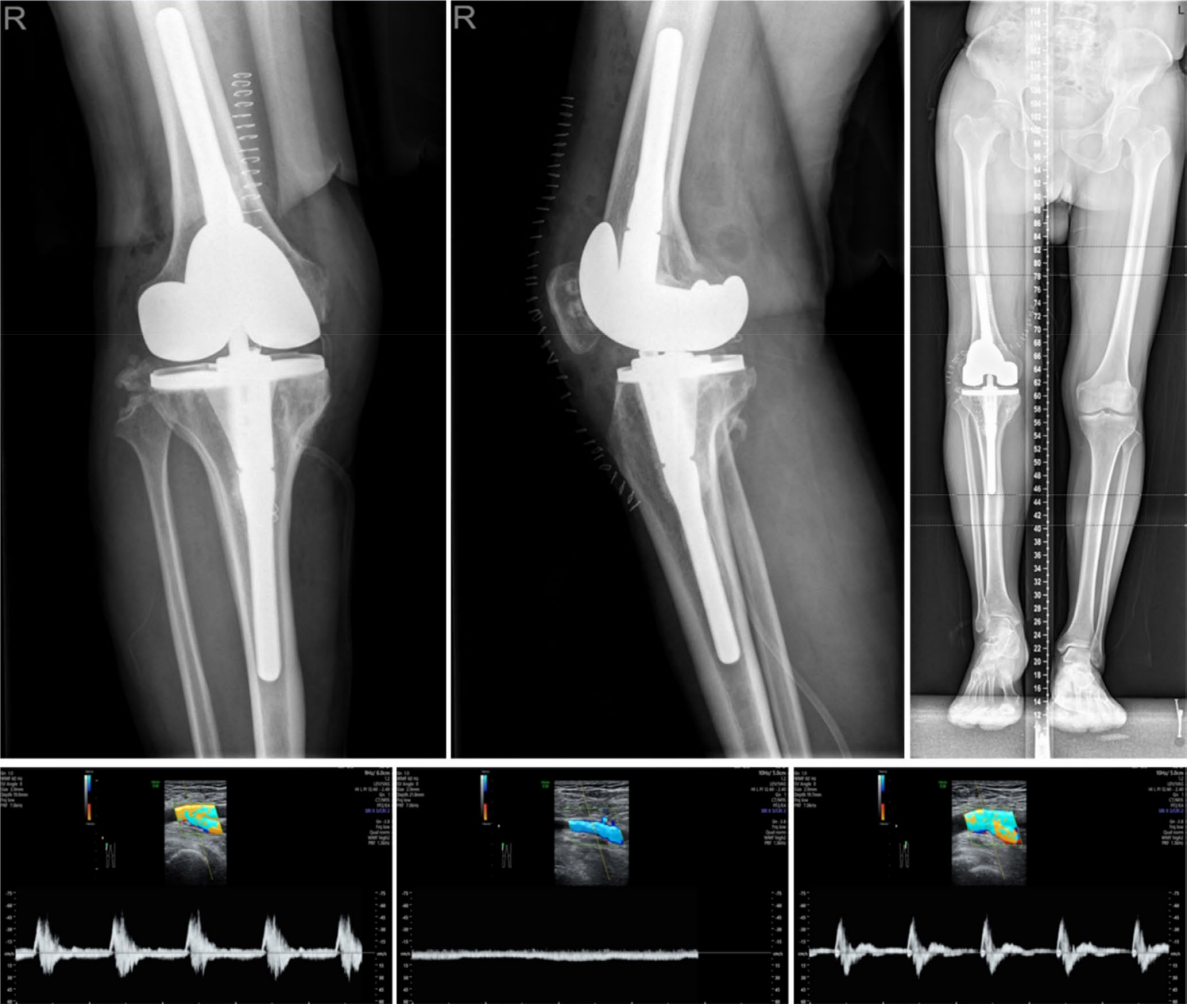
(A) X‐rays of post right TKR. Status post right knee total replacement shows acceptable implant alignment, size, and position. There are no fractures, translations, or dislocations. Moderate joint effusion is present. Multiple skin staples, soft tissue edema, and air foci are consistent with early postoperative changes. (B) Doppler ultrasound of the arteries and veins in both lower limbs after TKR reveals a normal inner diameter of the arteries in both the right and left lower limbs, with thickened and uneven intima. Multiple small, strong light spots can be seen on both arterial walls, accompanied by comet tails. There is no stenosis in the arterial lumen, and Doppler blood flow filling in the arteries is good.

The antibiotic Cefuroxime Sodium 1.5 g bid, anticoagulant Nagroparin Calcium 0.4 mL, and analgesic Flurbiprofen Axetil 50 mg bid were used postoperatively. The patient refused to use the analgesic pump following the surgery. The postoperative drainage volume was recorded by nurses after surgery. The total drainage volume was 320 mL on the first day after the surgery. The patient's condition remained stable, the postoperative X‐ray showed that the prosthesis was in place (Figure 2A), and the ultrasound of the arteries and veins of both lower extremities showed that some of the right and left lower extremities had atherosclerosis with multiple small plaques (Figure 2B). On the second day, the cotton pad dressing was removed, and exercise was initiated. The amount of drainage decreased to 220 mL on the second day and to 120 mL on the third day.

**FIGURE 3:**
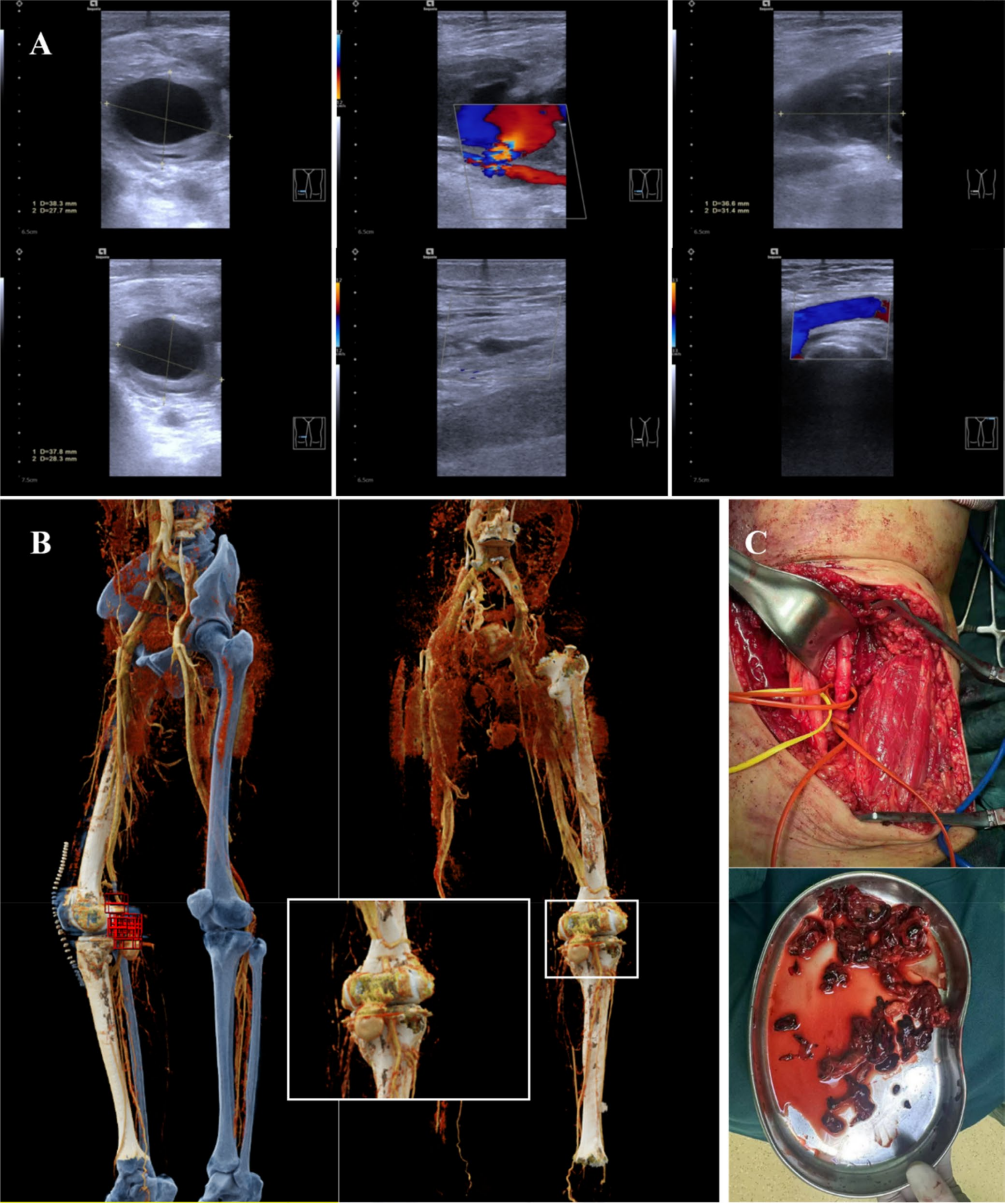
(A) Ultrasound examinations of lower limb vascular structures. A huge mixed‐echo mass was observed at the right popliteal fossa, with a long diameter that could not be measured. The larger transverse plane was approximately 3.78 × 2.83 cm, and the interior contained a dark area. Multiple low echoes were visible at the edges, with a large range of about 3.83 × 2.77 cm. A rupture could be seen in the deep plane connected to the popliteal artery. (B) CTA of the right knee after TKR. Following the bilateral replacement of the right knee joint, metal artifacts interfered with the imaging, making the local display of the popliteal artery unclear. A mass resembling a contrast agent overflow shadow was observed in the popliteal fossa, with a maximum cross‐section of about 32 × 23 mm, locally connected to the popliteal artery. (C): The pseudoaneurysm observed during vascular surgery.

On the fourth day, the patient had no significant discomfort after the drainage tube was removed when the drainage volume within 24 h was about 50 mL. Postoperative rehabilitation exercises were conducted by our hospital's rehabilitation doctors using the lower limb joint rehabilitation device CPM‐F (YTK‐F, Zhengda Medical, China). The patient was passively flexed at the knee for 1 h while lying flat, without any manual pressure applied. The flexion angle on the first day was 0°–90°. The patient suddenly experienced a tearing‐like pain during knee flexion exercise, followed by gradually increasing intolerable swelling pain behind the knee joint. Pain relief and other symptomatic treatments were administered immediately, and an appointment was scheduled for a lower limb vascular ultrasound (Figure 3A).‌ The radiology and vascular surgery departments were contacted to conduct a vascular CT angiography (CTA) (Figure 3B). Subsequently, the vascular surgery department performed vascular exploration and resected the pseudoaneurysm (Figure 3C).

Surgical dissection revealed the sciatic nerve, posterior tibial nerve, common peroneal nerve, and popliteal vein, all of which were protected by suspension with rubber slings. Exploration revealed a 5 cm diameter fluctuating mass in the popliteal fossa, along with pulsations in the proximal superficial femoral artery. After administering 2000 units of regular heparin intravenously, the proximal end of the popliteal artery was occluded with forceps, the wall of the pseudoaneurysm was incised, and a large amount of dark red thrombus was removed.

### 2.4 Further Follow‐Up

**FIGURE 4:**
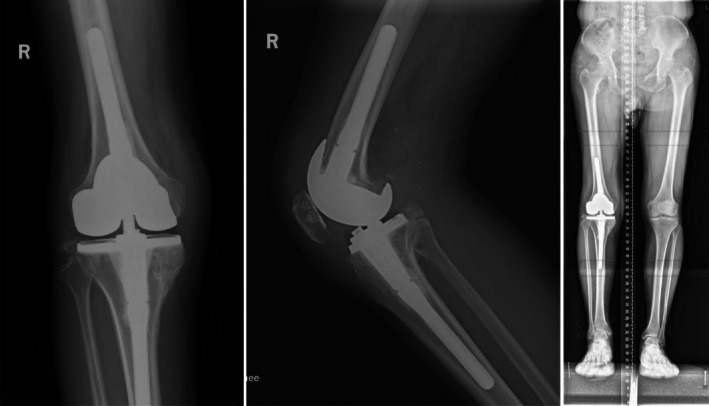
X‐rays of the follow‐up visit 1 month after discharge.

The patient underwent evaluation after surgery. He felt comfortable, the swelling had subsided, and the patient's thigh circumference had also improved. Nonetheless, owing to irritation of the posterior muscle group in his lower leg, he experienced restricted dorsiflexion of the ankle joint, a condition also evident in the postoperative full‐length radiograph of his lower limb (Figure 2). Following intensive postoperative exercise rehabilitation, the patient's ankle dorsiflexion notably improved. At the one‐month post‐discharge follow‐up, he reported that his knee joint could easily flex beyond 90° (Figure 4). Due to the patient feeling good knee joint function after surgery and not returning to the hospital for follow‐up, we contacted the patient by phone for long‐term functional follow‐up 1 year after surgery. The patient reported no significant pain and was able to walk 2 km. With external assistance, the knee joint can bend 120°.

On the X‐ray taken 1 month later, the patient's knee joint prosthesis was in place without any signs of looseness, and the knee joint exhibited a good range of motion. Additionally, it was noted that the patient's ankle range of motion had improved while standing, in contrast to the limited range observed when standing on tiptoe after surgery.

## 3 Discussion

### 3.1 Main Findings

This article reported a rare case of popliteal artery pseudoaneurysm caused by acute rehabilitation after total knee arthroplasty. The patient underwent lower limb ultrasound examination both before and after surgery, and the presence of an aneurysm was immediately detected during postoperative pain.

### 3.2 Comparison With Previous Literature

Pseudoaneurysm of the popliteal artery following knee arthroplasty represents an infrequent complication characterized by subtle symptoms, including concealed pain in the lower extremities, which can be easily mistaken for postoperative pain. This misinterpretation may result in delayed diagnosis and treatment. We conducted a review of recent case reports concerning pseudoaneurysm of the popliteal artery post‐knee arthroplasty. Aside from cases involving intraoperative bleeding, a minority were attributed to trauma, while the remaining reports did not specify particular etiologies. As illustrated in Table 1, the challenges associated with detecting this condition, coupled with the potential for delayed symptom onset, can result in prolonged periods of undiagnosis, complicating the identification of its etiology. In our case, we promptly identified this situation, providing the possibility for clarifying the etiology of pseudoaneurysm formation.

**TABLE 1:** Literature review of case reports on popliteal artery pseudoaneurysms after total knee arthroplasty.

Authors and year	Age (years)/gender	Time when symptoms began	Symptoms	Causes	Time of diagnosis
O'Connor JV 1998 [11]	71/M	9 days	Increasing left calf pain with swelling and difficulty ambulating	—	4 months
Karkos CD 2000 [12]	72/F	3 weeks	A pulsatile swelling and an audible bruit in the popliteal fossa	—	7 weeks
Hartford JM 2002 [13]	42/F	1 year	Massive swelling, ecchymosis, and a pulsatile mass behind her knee	After twisting her knee	1 year
Ibrahim M 2004 [14]	71/M	1 month	Ecchymosis of the left knee and calf, with calf swelling and tenderness	—	1 month
D'Angelo F 2007 [15]	52/F	—	A massive hemorrhage from the popliteal pit	—	During knee arthroplasty procedures
Behnke NM 2007 [16]	58/M	1 year	Acute thigh swelling	Infection	1 year
Sandoval E 2008 [17]	68/F	2 days	An acute onset of increasing pain in left popliteal fossa. Swelling and tenderness in the popliteal fossa, with a local hematoma.	—	2 days
Cowell GW 2009 [18]	87/M	5 days	Leg swelling, bruising, and pain	—	24 days
Sloan K 2009 [19]	70/M	After surgery	Left calf pain and swelling	—	—
	65/F	After surgery	Extensive bruising and swelling of the right calf associated with a cold pale left foot	—	—
De Haro Miralles J 2010 [20]	78/F	5 days	Pain in the right calf that increased when she walked, swelling and oedema in the popliteal fossa	Walk	5 days
Geertsema D 2012 [21]	69/F	6 days	progressive pain, swelling, and tightness of the right calf	—	6 days
	60/M	5 days	Progressive swelling, pain, and tightness of the left lower leg	—	21 days
	67/F	2 days	Pain at the medial part of the left ankle and plantar sensory loss of the foot	—	1 month
Schouten Van Der Velden AP 2012 [22]	61/M	After surgery	Severe pain	—	17 days
De Santis F 2013 [23]	76/F	9 days	A calf haematoma and large retro‐articular pseudoaneurysm	—	9 days
Boutchichi A 2013 [24]	64/M	After surgery	A recurrent knee haemarthrosis	—	6 weeks
	73/M	18 days	A posterior throbbing pain, a stiff knee and a functional disability	—	18 days
	66/F	After surgery	Calf pain and foot hypoaesthesia	—	6 weeks
González Rodríguez JC 2012 [25]	80/F	After surgery	Widespread hematoma of the skin, edema and swelling	—	8 days
Tejero‐Garcia S 2014 [26]	69/F	12 h	Pain and swelling	—	29 days
Ghazala CG 2015 [27]	72/M	3 weeks	A foot drop	—	5 weeks
Reynolds A 2017 [28]	72/M	After surgery	The left leg was cool from below the knee	—	2 days
Alammari N 2024 [29]	61/M	10 days	Left leg pain, numbness, and swelling, mainly in the calf muscle	—	17 days
Dincal T 2025 [30]	81/F	1 day	Discomfort in the lower extremities and posterior knee pain	—	1 day

### 3.3 Initiating Rehabilitation Promptly After Surgery

Early postoperative exercise is commonly considered beneficial for rapid recovery. However, our article reported a case of exercise‐related postoperative pseudoaneurysm. Early exercise following total knee arthroplasty (TKA) is crucial for optimizing recovery and enhancing functional outcomes. Research indicates that immediate postoperative interventions, including specific exercise regimens, significantly improve knee range of motion (ROM) and muscle strength [31, 32, 33]. For instance, a study demonstrated that patients who participated in early rehabilitation exercises exhibited better knee flexion and clinical outcomes compared to those who did not participate [33]. This underscores the importance of initiating rehabilitation promptly after surgery to mitigate the loss of muscle strength and function often associated with major surgical procedures. In addition to physical exercises, the timing and intensity of rehabilitation are crucial in determining recovery outcomes. A meta‐analysis has revealed that physiotherapy exercises initiated too late or performed with inadequate intensity may fail to produce the desired benefits in post‐operative recovery [34]. Therefore, it is imperative for clinicians to devise rehabilitation programs that are not only started early but also customized to address the unique needs of each patient, guaranteeing both the effectiveness and safety of the exercises.

### 3.4 Postoperative Non‐Acute Period Rehabilitation

Early exercise is generally encouraged after surgery [35], but special caution is needed for certain patients, particularly those with unique medical histories or conditions. In this specific case, the patient had old traumatic arthritis and had undergone internal fixation surgery on the tibial plateau. Due to severe adhesion of blood vessels to soft tissues and poor preoperative mobility, his tissue structure differed from the norm. We speculated that the pseudoaneurysm of this patient might be related to the process of rupture of the blood vessel wall caused by repeated stretching of blood vessels in scar tissue during early rehabilitation on the fourth day after surgery. In contrast, almost all other patients experienced no issues starting exercise during the acute phase, as maximum knee flexion was achieved during surgery. Additionally, rigorous hemostasis was achieved at the posterior aspect of the knee during the procedure, resulting in no significant postoperative bleeding.

Therefore, the indirect cause can be attributed to the concealed vascular injury that occurred during the operation. Potential reasons for this injury, which resulted in damage to the blood vessel, could include direct damage inflicted by the oscillating saw due to unclear soft tissue boundaries; inadvertent pulling on the popliteal artery while using a retractor; or thermal damage to the blood vessel caused by the close proximity of bone cement to the surrounding soft tissue [29].

A recent review suggests that there are no standardized postoperative limitations following TKA, and the return to preoperative activities should be based on each individual's competency, incorporating methods to minimize high‐impact stress on the joint [36]. Perhaps allowing patients a specific period of recovery time after surgery to repair any damage to the vascular endothelium sustained during the procedure could help prevent the development of pseudoaneurysms.

In the context of early functional exercise following total knee replacement (TKA), the mechanisms behind vascular injury, particularly involving the popliteal artery, necessitate thorough consideration [37]. As patients undergo rehabilitation, the heightened mechanical demands placed on the knee joint can unintentionally result in localized vascular stress and trauma. The increasing incidence of knee arthritis in the affected population has led to a surge in surgical interventions, which, while improving outcomes, also introduces a range of complications, including vascular injuries [38]. Early resumption of functional activities, intended to facilitate recovery, may inadvertently exacerbate these injuries, especially in individuals with pre‐existing vascular compromise. For patients with significant preoperative tissue adhesion, delaying exercise rehabilitation until the non‐acute postoperative period may be a more prudent choice for ensuring safety.

### 3.5 Strengths and Limitations

The advantage of this study is that in addition to the literature review, the manuscript also describes the pseudoaneurysm of the popliteal artery that occurred on the fourth day after total knee replacement (TKR), which is related to the time of early knee flexion rehabilitation. However, there are several limitations to our study. First, although the pseudoaneurysm is speculated to be caused by rehabilitation training, the possibility of intraoperative potential vascular injury (such as thermal damage from bone cement or retractor traction) is not excluded, leading to a weak causal inference. Second, in our review, we listed whether patients had lower limb activity after surgery to compare the causes of pseudoaneurysms. Most reports did not mention the patients' postoperative rehabilitation exercises, making it impossible to conduct comparisons. As illustrated in Table 1, the challenges associated with detecting this condition, coupled with the potential for delayed symptom onset, can result in prolonged periods of undiagnosis, complicating the identification of its etiology.

## 4 Conclusion

This case highlights the importance of adopting a cautious approach to post‐operative rehabilitation following total knee replacement, with particular attention to the risk of pseudoaneurysm formation in the popliteal artery. This emphasizes the need for individualized rehabilitation protocols that strike a balance between the benefits of early mobility and the potential for adverse events, ultimately enhancing patient safety and outcomes during the recovery period. Future research is crucial to refine these protocols and further elucidate the mechanisms underlying such vascular complications.

## Author Contributions

**Jiajiang Jiang:** project administration, resources, supervision. **Shigui Yan:** visualization. **Sihao Li:** methodology, writing – review and editing, project administration, resources, supervision, formal analysis, visualization. **Haobo Wu:** validation, visualization, methodology. **Xiang Zhao:** writing – original draft, conceptualization, investigation, software, data curation, formal analysis, supervision, funding acquisition.

## Funding

The authors have nothing to report.

## Ethics Statement

The patient's medical history and design comply with the principles of the Helsinki Declaration, fully respecting the informed consent rights of the patient and their family. The ethical review approval 2025‐1117 from the Human Research Ethics Committee of the Second Affiliated Hospital of Zhejiang University School of Medicine.

## Consent

Written informed consent has been obtained from the patient to publish this paper.

## Conflicts of Interest

The authors declare no conflicts of interest.

## Data Availability

The data that support the findings of this study are available from the corresponding author upon reasonable request.
